# Adoption of Machine Learning Algorithm-Based Intelligent Basketball Training Robot in Athlete Injury Prevention

**DOI:** 10.3389/fnbot.2020.620378

**Published:** 2021-01-15

**Authors:** Teng Xu, Lijun Tang

**Affiliations:** ^1^Department of Physical Education, Shanghai Jiao Tong University, Shanghai, China; ^2^Physical Education College, Shanghai Normal University, Shanghai, China

**Keywords:** machine learning algorithm, intelligent robot, basketball training, sports injury, athlete injury prevention

## Abstract

In order to effectively prevent sports injuries caused by collisions in basketball training, realize efficient shooting, and reduce collisions, the machine learning algorithm was applied to intelligent robot for path planning in this study. First of all, combined with the basketball motion trajectory model, the sport recognition in basketball training was analyzed. Second, the mathematical model of the basketball motion trajectory of the shooting motion was established, and the factors affecting the shooting were analyzed. Thirdly, on this basis, the machine learning-based improved Q-Learning algorithm was proposed, the path planning of the moving robot was realized, and the obstacle avoidance behavior was accomplished effectively. In the path planning, the principle of fuzzy controller was applied, and the obstacle ultrasonic signals acquired around the robot were taken as input to effectively avoid obstacles. Finally, the robot was able to approach the target point while avoiding obstacles. The results of simulation experiment show that the obstacle avoidance path obtained by the improved Q-Learning algorithm is flatter, indicating that the algorithm is more suitable for the obstacle avoidance of the robot. Besides, it only takes about 250 s for the robot to find the obstacle avoidance path to the target state for the first time, which is far lower than the 700 s of the previous original algorithm. As a result, the fuzzy controller applied to the basketball robot can effectively avoid the obstacles in the robot movement process, and the motion trajectory curve obtained is relatively smooth. Therefore, the proposed machine learning algorithm has favorable obstacle avoidance effect when it is applied to path planning in basketball training, and can effectively prevent sports injuries in basketball activities.

## Introduction

Basketball is widely accepted as one of the world's top sports. In the process of training, there are more comprehensive requirements on the physical and competitive ability of athletes (Coglianese and Lehr, 2017; Liu and Hodgins, 2018; Ji, 2020). Sports injury is an inevitable problem faced by athletes in all sports, and it is also an unavoidable situation in training and competition. Basketball increases the risk of injury due to the high intensity and high-speed collisions between players in the sport. Especially, excessive use of joints, muscles, and ligaments will lead to joint sprains and muscle strains (Li et al., 2016). Sports injuries not only damage the physical and mental health of individual athletes, but also affect their competitive ability and restrict the development of the whole team (Li et al., 2019). To prevent sports injuries caused by collisions in training or competitions, it is necessary to optimize the offensive and defensive paths by intelligent basketball training robots.

In modern basketball, the advantages of intelligent robots based on artificial intelligence, machine learning, and other algorithms in sports training gradually appear (Zhao et al., 2018). In the context of the Internet of Things, Lv (2020) combined high-tech achievements in multiple fields to realize human-computer interaction in a natural and intelligent way. Information processing and operation through computer programs can form an interactive and simulated natural state and three-dimensional environment on the display terminal, which can make people feel immersed. This is also of great reference value for intelligent basketball training (Lv, 2020). Toyota has developed a basketball robot called “Cue,” with the internal visual feedback system as its core. Before shooting the shot, “Cue” will determine the 3D image of the surrounding environment via its limbs and torso, calculate the height and force of the basketball through the algorithm, and thus achieve the goal of accuracy (Narayanan et al., 2020). Shooting results show that it is considerably more accurate than professional basketball players, because it can continue to learn and improve itself under strong support of artificial intelligence technology. Some scholars combined the labVIEW software development platform and visual development module of the American company. In relative motion state, the basketball could achieve the goal of precise positioning and meet the adoption requirements of basketball robots in actual games (Bing et al., 2020). At present, there is an ultrasonic-based multi-degree-of-freedom basketball robot complex path tracking control system. Ultrasound can accurately provide the distance information of obstacles encountered by the robot. If there is an obstacle, the received information is converted and fed back to the main control board in the form of an electrical signal, which ultimately effectively improves the recognition rate of the basketball robot to the obstacle. Since most sports injuries in basketball activities are caused by failure to avoid obstacles and errors in cooperation between attack and defense, the machine learning algorithm is utilized to plan the movement posture and autonomous path of basketball players, so as to effectively avoid obstacles and prevent sports injuries.

In this study, a robot system for basketball training based on machine learning algorithm was proposed from the perspective of preventing athletes from training injuries and the characteristics of fierce confrontation in basketball. The autonomous path planning problem of the robot system applies the improved Q-Learning algorithm. On this basis, combined with the basketball trajectory model, the motion robot controller system based on fuzzy control principle was analyzed. Finally, the feasibility of the improved algorithm to solve the autonomous motion was verified through simulation experiments. According to the obstacle avoidance ability of the sports robot based on machine learning algorithm in basketball training, the occurrence of sports injuries was effectively prevented.

## Materials and Methods

### Sport Recognition in Basketball Training

**Figure 1:**
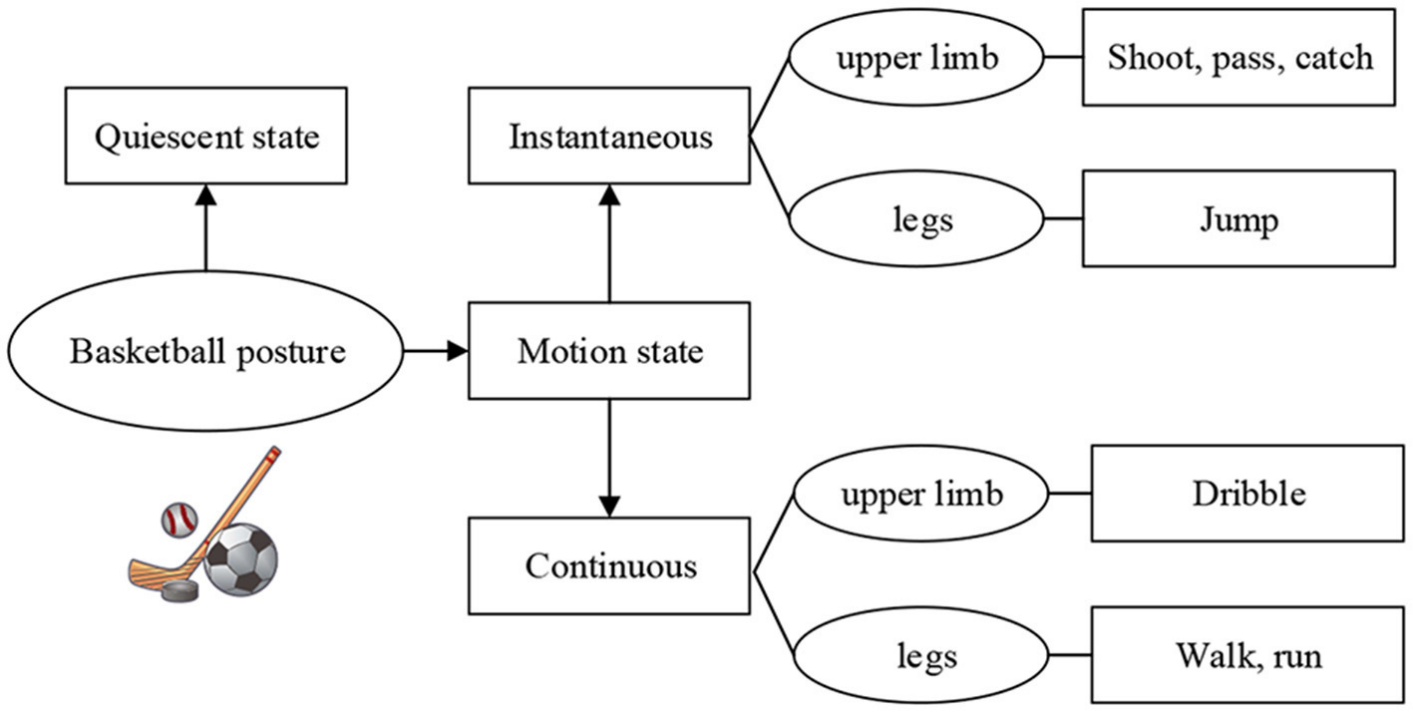
The composition of the sports posture of basketball training.

The movements involved in basketball constitute a relatively complex and comprehensive movement system. The recognition of basic basketball movements is of great value to further improve the training efficiency of athletes, and the division of basketball posture composition is shown in Figure 1. In ordinary training, basic training movements mainly include dribbling, ball control, passing, catching, shooting, and pace adjustment (Lobos-Tsunekawa et al., 2018). In specific training, basketball posture can be divided into two states: static and moving based on the different states of the body. The basketball action can also be divided into two kinds according to whether it is periodic or not: instantaneous and continuous. During continuous movement, the athletes' upper and lower limbs keep a continuous periodic transformation. Therefore, unit actions can be divided according to specific action data (Chau et al., 2019; Yoon et al., 2019; Stübinger et al., 2020). When the body movement is described, angular velocity can be taken as the reference base of data division due to its intuitive advantage of data.

After data division, the unit action data composed of acceleration and angular velocity can be obtained. The acceleration vector sum and the angular velocity vector sum are expressed as *a*_*n*_ and *g*_*n*_, respectively.

(1) $$\begin{array}{l} a_{n}=\sqrt{{\left(a_{n}^{x}\right)}^{2}+{\left(a_{n}^{y}\right)}^{2}+{\left(a_{n}^{z}\right)}^{2}} \end{array}$$

(2) $$\begin{array}{l} g_{n}=\sqrt{{\left(g_{n}^{x}\right)}^{2}+{\left(g_{n}^{y}\right)}^{2}+{\left(g_{n}^{z}\right)}^{2}} \end{array}$$

where, $a_{n}^{x}$, $a_{n}^{y}$, and $a_{n}^{z}$ represent the acceleration of the three axes of the *n*^*th*^ sampling point *x, y*, and *z*, respectively; and $g_{n}^{x}$, $g_{n}^{y}$, and $g_{n}^{z}$ represent the angular velocity of the three axes of the nth sample point *x, y*, and *z*, respectively.

Each unit action was taken as a sample in this study. Among them, N represents the number of sampling points in each unit action, then each sample is a dimensional matrix (the composition of the 8-dimensional vector includes the acceleration and angular velocity of the three axes, as well as the combined acceleration and the combined angular velocity). The calculation of the mean value and variance of a certain component of the acceleration in the unit action are as Equations (3) and (4), respectively.

(3) $$\begin{array}{l} \mu_{a}=E\left(a\right)=\frac{1}{N}\sum_{i=1}^{N}a_{i} \end{array}$$

(4) $$\begin{array}{l} \delta^{2}=\frac{1}{N}\sum_{i=1}^{N}{\left(a_{i}-\mu_{a}\right)}^{2} \end{array}$$

where, *a* represents a certain component of acceleration.

The signal is converted from the time domain to the frequency domain through discrete Fourier transform, Fourier transform result *S*_*DFT*_ (*n*) of the *n*^*th*^ sampling point is calculated as Equation (5), and the corresponding frequency *f* after Fourier transform is calculated as Equation (6).

(5) $$\begin{array}{l} S_{DFT}\;\left(n\right)=\sum_{i=1}^{N}a_{i}e^{-j\frac{2\pi }{N}in} \end{array}$$

(6) $$\begin{array}{l} f=k\times \frac{f_{s}}{N} \end{array}$$

where, *j* represents the imaginary unit, *k* represents the sampling point corresponding to the peak value of the Fourier transform, and *f*_*s*_ represents the sampling frequency of the sensor.

After a series of operations such as data collection, preprocessing, and feature extraction, a set of feature vectors describing basketball actions can be obtained, and the classification to which these abstract features belong can be obtained using the classifier model (Hildebrandt, 2019).

**Figure 2:**
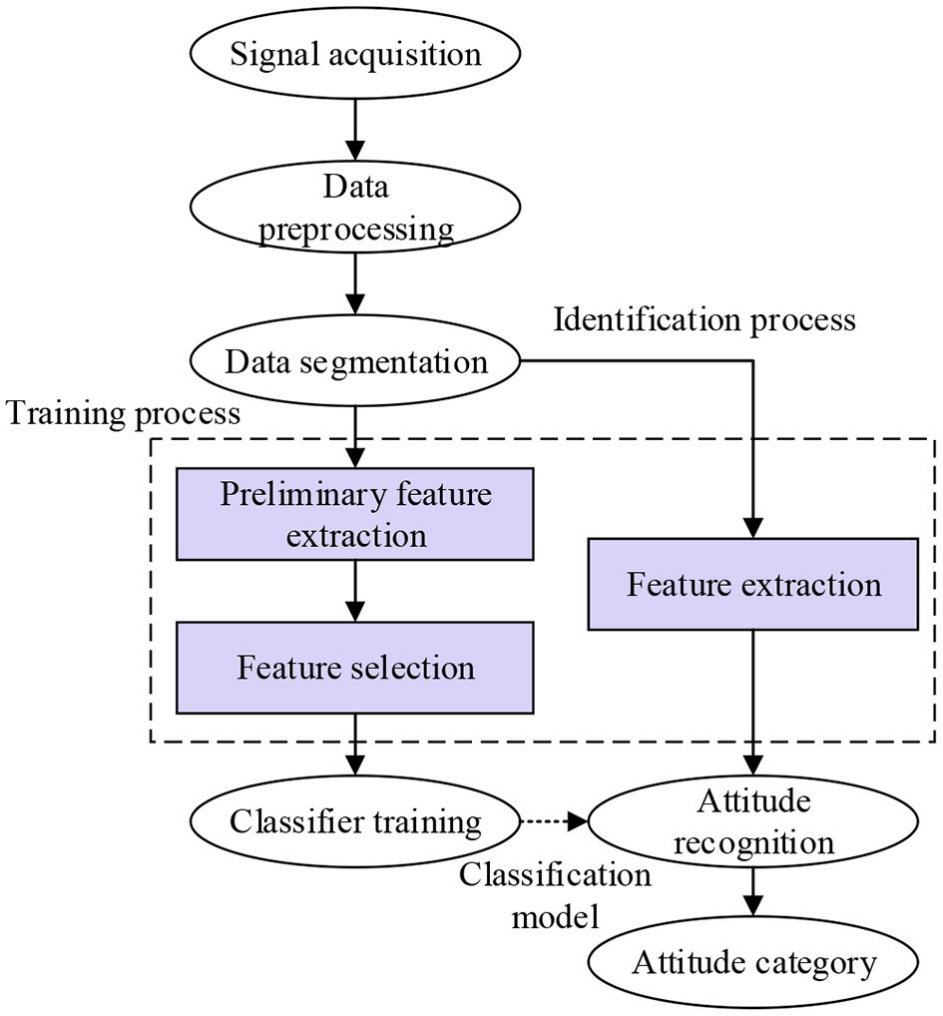
The process of basketball sport recognition.

The process of basketball sport recognition mainly includes data acquisition, data preprocessing, data division, feature extraction, and classifier training, as shown in Figure 2. During data acquisition, the physiological signal or physical signal of individual athletes is generally collected through sensor equipment. During data preprocessing, the data collected are desiccated and normalized to obtain more accurate signals. During data partition, the data extraction of single action in time domain and frequency domain is realized, and the data characteristics are analyzed separately. In the next step of feature extraction, the unit action is analyzed, and the attribute features are extracted and taken as samples. At the final classifier stage, the sample data are constructed into a classification model according to different classification principles to complete the classification of the sample data (Mejia-Ruda et al., 2018; Mullard, 2019; Starke et al., 2020).

### Intelligent Path Autonomous Planning Based on Machine Learning Algorithm

**Figure 3:**
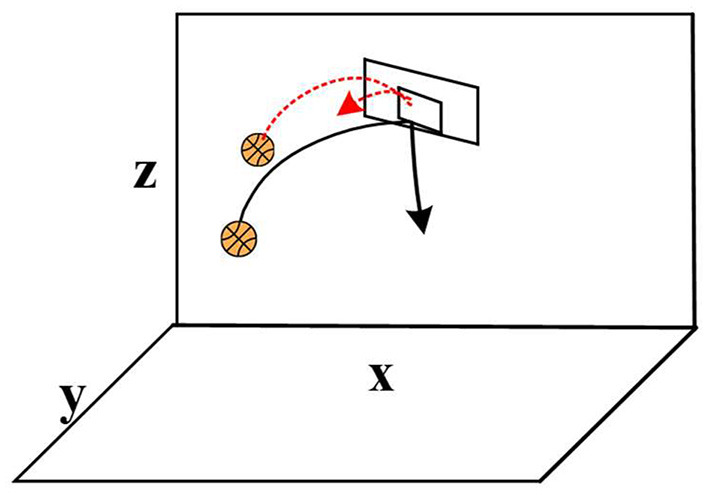
Correct and incorrect basketball path planning results.

From the perspective of sports cognition, the decision-making of basketball players directly affects the tactical performance and game result of the whole team (Žemgulys et al., 2018; Kim and Lee, 2020). Therefore, players are required to be able to capture the basketball target in real time and complete relevant information processing when make decisions. Not only do they need to lock in the basketball target that is active, but they also need to make decisions as quickly as possible. Planning the trajectory of real-time change can improve the hit rate. The shooting action was taken as an example in this study, and a clear route should be established before athlete shot a basketball shot. With the parabolic form taken as the premise, a reasonable angle needs to be adjusted in the global path planning (Pan and Li, 2020). The correct and incorrect basketball path planning results are shown in Figure 3.

The learning system mainly implements the decision-making and motion planning of the intelligent basketball robot through environmental map construction and path planning. Machine learning includes supervised learning, unsupervised learning, and reinforcement learning (Zhu et al., 2019; Zhang et al., 2020). Among which, reinforcement learning emphasizes how to evaluate selection actions based on the environment to maximize the expected benefits. Through continuous trial and error, the system gradually improves the action selection strategy, and finally achieves the purpose of learning. Q-Learning learning algorithm is one of the most widely used important algorithms in reinforcement learning. A robot based on the Q-Learning algorithm only knows the set of actions that can be selected at the moment. To represent the action reward value from one to the next state, a corresponding matrix is usually constructed, from which the Q matrix that can guide the robot's activities is obtained (Hung et al., 2018; Tieck et al., 2019; Wong et al., 2019).

First, the value *Q(s,a)* should be initialized. In the current state, the robot selected a strategy according to the action, the next state was obtained after the strategy-guided exercise, and then the above selection process was repeated according to the *Q(s,a)* value corrected by the update rule until the end of the learning. The Q-Learning algorithm needed to choose through continuous trial and error and action selection. Therefore, only through continuous correction of feedback information could the final suitable strategy be obtained, and that's why the algorithm had slow update speed (Sombolestan et al., 2019).

In the Q-Learning algorithm, the ε-greedy strategy was utilized to balance the exploration, but the value of the exploration factor ε would decrease as the algorithm training time increased (Maryasin et al., 2018). The purpose of balancing the exploration process was obtained by dynamically updating the value of the exploration factor ε. The number of successful path-finding and the number of different paths found by the robot were taken as the basis for controlling the ε value. The robot had a low number of successful pathfinding initially, so it should maintain a high ε value at the beginning and keep trying new actions. When the robot was gradually familiar with the environment, the number of successful pathfinding would exceed the threshold, and then the robot state action value function would stabilize.

When the Q-Learning algorithm was applied to the robot path planning, in the initial state *s*_*t*_, a certain action *a*_*t*_ was selected according to the strategy; when the state was transferred to the state *s*_*t*+1_, an immediate return could be obtained. The Q reality can be expressed as Equation (7).

(7) $$\begin{array}{l} Q\left(s_{t},a_{t}\right)=r_{t+1}+\gamma \;\underset{\alpha }{max}\;\left(s_{t+1},a_{t+1}\right) \end{array}$$

When the robot chose the next state, the probability method could be adopted to select the action to avoid maximizing the deviation. The calculation of probability is expressed as Equation (8).

(8) $$\begin{array}{l} P\left(s|a_{k}\right)=\frac{Q\left(s,a_{k}\right)}{\underset{j}{\sum }Q\left(s,a_{j}\right)} \end{array}$$

As for the slow update speed of Q-Learning algorithm, a counting threshold was supplemented. The update of the Q value was determined according to the cumulative access times to the “state-action pair” <s, a>. When the cumulative access times reached the threshold, the Q value of “state-action pair” would be updated to improve the efficiency of the algorithm.

### Basketball Trajectory Model

A key indicator for evaluating basketball players is shooting accuracy, which is also the primary issue for most basketball players to improve their competitive ability. If the basketball movement is quantified and the data of basketball movement is calculated from the perspective of mechanics, the athletes can be trained scientifically according to the numerical results (Gonzalez et al., 2018). This not only improves the training efficiency of athletes, but also avoids fatigue sports injuries caused by additional training. In the shooting action, the basketball shot from the optimal shooting corner not only consumes the least effort, but also the basketball has the farthest flight distance and the highest scoring rate (El-Shamouty et al., 2019). In basketball training, if athletes can find their optimal shooting angle and form muscle inertia through repeated training, the athlete's training level can be greatly improved.

The main external influence on basketball trajectories was air resistance, so the basketball trajectory in the absence of is mainly analyzed in this study. The basketball and the athletes with arms outstretched was taken as a whole for measurement. Among this, *x* and *y* represent the uniform movement in the horizontal and vertical directions, respectively, and the Equation (9) can be obtained.

(9) $$\{\begin{array}{l} x=vt\cos\alpha \\ y=-gt/2+vt\sin\alpha \end{array}$$

where, *v* represents the speed when the basketball is shot, and α represents the angle when the basketball is shot.

The common diameter standard for basketball is 24.6 cm, the diameter of the standard basket is 0.45 meters, and the basket is 3.05 meters above the ground. The backboard specifications are 1.80 meters in length, 1.05 meters in vertical height, and 0.03 meters in thickness. The horizontal distance from the center of the basket is 4.21 m for free throws, and 6.25 m for three-pointers. On the free throw line, the optimal shooting angle and the minimum shooting speed can be calculated according to the height of the basketball shot and the vertical distance needed to move, as shown in Table 1.

**Table 1:** The best shot angle and minimum shot speed for different shot heights and vertical distances.

**Basketball shooting height (m)**	**Vertical distance needed to move (m)**	**Minimum release angle (°)**	**Minimum release speed (m/s)**
2.89	0.16	46.09	6.55
2.68	0.37	47.58	6.72
2.25	0.80	50.49	7.06
2.06	0.98	51.56	7.21
1.94	1.10	52.26	7.31
1.76	1.29	53.55	7.45
1.72	1.34	53.78	7.50

It is obvious that the optimal shooting angle of the basketball decreases with increasing shooting height and increases with increasing shooting speed. Therefore, for the taller players, the basketball shooting angle or speed should be reduced appropriately during the jump shot. By shortening the flight arc of basketball and reducing the external influence of environmental factors as much as possible, the players can improve their shooting percentage. In addition, due to the increase in the height of shooting, the optimal shooting angle of basketball has a great change. In the jump shot training, the sensitivity of the athlete's fingers should be improved.

### Design of Obstacle Avoidance Algorithm Based on Fuzzy Control of Motion Robot

Fuzzy control simulates human behavior and then makes decisions based on fuzzy reasoning. The core of fuzzy control is fuzzy controller, which is mainly responsible for fuzzy processing of input variables, rule design and reasoning, and de-fuzzy. Generally, a complete fuzzy controller consists of four modules: fuzzy interface, knowledge base, reasoning mechanism, and de-fuzzy. The precision of the fuzzy controller completely determines the performance of the entire fuzzy control system to a certain extent (Alonso-Martín et al., 2017). First of all, the precise amount of input can only be utilized by the fuzzy controller after the fuzzy transformation of the variable. The finer the fuzzy subset division, the more the fuzzy rules. The inference machine realizes fuzzy inference by selecting a certain inference algorithm and combining fuzzy rules, and finally completes the output of the control quantity. After fuzzy reasoning, a fuzzy set can be obtained, and it can be applied by the control system only by defuzzing the set into precise values.

**Figure 4:**
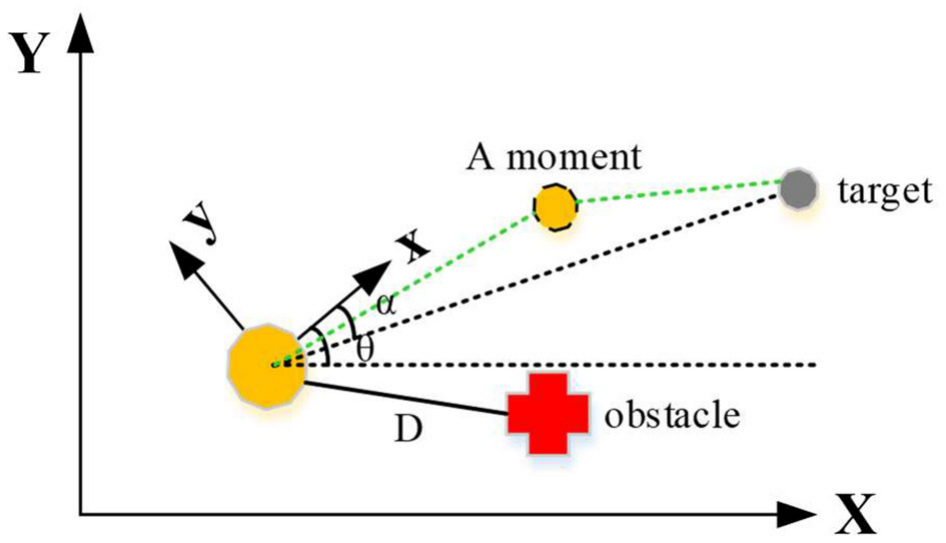
The coordinate system of the moving robot system.

The control structure of the moving robot designed in this work is divided into two parts, that is, to approach the target point and to avoid obstacles. First, a two-dimensional coordinate system is established for the mobile environment of the moving robot, including global coordinate system and local coordinate system, as shown in Figure 4. The distance between the robot and the target point is *D*, the direction angle of the robot is θ, and the direction angle between the robot and the target point is α.

The activities of a moving robot are divided into two kinds: the obstacle-free tendency behavior and the obstacle-avoiding behavior, both of which are realized by fuzzy controller. During the barrier-free approach to the target point, the distance of the robot from the target point, the difference between the direction angle of the mobile robot and the direction angle of the target point, and the difference between the heading angle and the direction angle of the target are taken as input, then the angular velocity and linear velocity of the robot are output. The linear speed and angle are controlled, so that the robot can constantly adjust posture and approach the target. In the obstacle avoidance behavior with obstacles, the ultrasonic signals acquired around the robot are taken as input to ensure that the robot can effectively avoid obstacles.

The obstacles are set to be at the right front and left front of the robot, respectively, and two fuzzy controllers are applied. When the robot detects an obstacle, it first judges the specific orientation and then chooses to enter the obstacle-avoidance fuzzy controller to calculate the speed that can avoid the obstacle. When the robot leaves the obstacle, it switches to the fuzzy controller which tends to the target point and moves toward the target. According to the fuzzy segmentation of each input variable, the fuzzy control rules are as follows. If the obstacle is at the right of the robot, it is set to turn counterclockwise. If the obstacle is at the left of the robot, it is set to turn clockwise. The linear velocity and angular velocity of the robot are affected by the distance from the obstacle. When the robot is far away from the obstacle, its output linear velocity and angular velocity are larger. When the robot is close to the obstacle, the output linear velocity and angular velocity are smaller.

**Figure 5:**
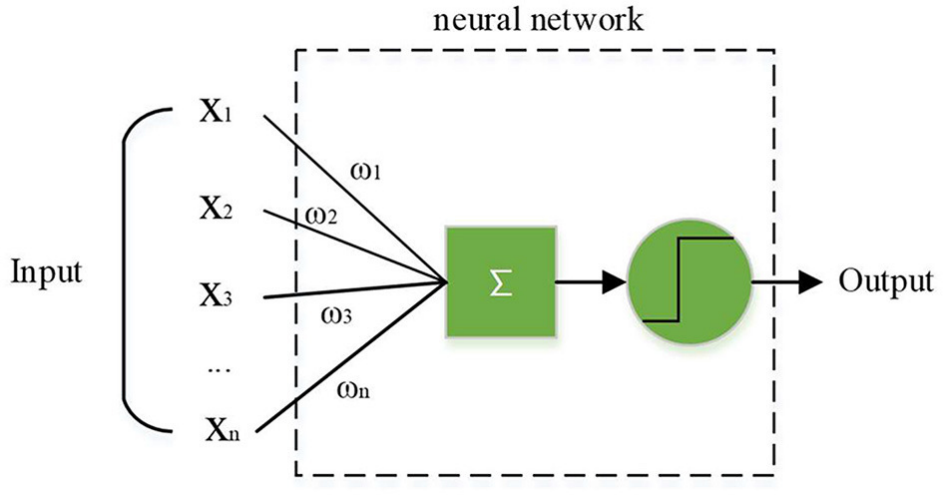
Artificial neuron model.

In the actual basketball game, since the defender is dynamic, dynamic obstacles should be adopted in the training strategy. An artificial nerve is an abstract model that can be implemented by a circuit or control program, as shown in Figure 5. In this model, neurons receive signals from more than one neuron and use them as input. After these signals are compared through the threshold, they are finally processed by the activation function to produce the output of the neuron (Fu et al., 2019; Lesort et al., 2020). Artificial neuron model can simulate the working principle of neuron cells, and get better decision-making data through constant intelligent training, so as to improve the decision-making efficiency and level.

### Robot Simulation Experiment

Windows 10 operating system and Inter Core I7-2600 were adopted for the simulation experiment in this study. The path planning algorithm of the robot system was written in Matlab programming language. The traditional Q-Learning algorithm adopted Markov decision process for modeling. The learning rate was initialized to 0.01, the deduction factor was set to 0.8, and the initial value of the exploration factor was set to 0.4. Adopting convolutional neural network to approximate the Q value function, that is, utilizing Q-Network to represent the Q value. The aim of marking the deviation value of the signal and the output of the convolutional neural network is to minimize the loss function. Therefore, a certain training sample is needed, including a large amount of labeled data. Then, backpropagation and gradient descent are adopted to update the parameters of the convolutional neural network. The experience playback is added. First, the sample information discovered by the system is stored in experience pool *D*. The sample information is a four-tuple consisting of the current state *S*_*t*_, the current state action value *a*_*t*_, the immediate reward *r*_*t*_ obtained by the current action, and the next state *S*_*t*+1_. During training, a set of samples is randomly selected from the samples stored in the experience pool *D* through the experience playback mechanism, and then the gradient descent method is adopted for iterative learning.

$s^{i}=\left(s_{1}^{i},s_{2}^{i},\ldots s_{n}^{i}\right)$ represents the obstacle avoidance path of the *i*-th robot. However, since the number of steps (length) of the path is different, the robot with few steps is expanded, and the target state is filled into the static obstacle avoidance path of a single robot. In addition, since the state vector is adopted in the multi-robot system, the static obstacle avoidance path of each robot is combined into a state vector for representation. First, the *Q* value is initialized to 0. When the multi-robot system performs $\left(s_{1}^{1},s_{1}^{2},s_{1}^{3}\right)$ to $\left(s_{2}^{1},s_{2}^{2},s_{2}^{3}\right)$, the action value function of state transition is set to a reasonable value >0. It can force the multi-robot system to have a certain understanding of the environment and tend to choose the optimal static obstacle avoidance path instead of randomly trying actions.

Besides, the 12 × 12 grid map was adopted, and the location of obstacles was random. The traditional Q-Learning algorithm and the improved Q-Learning algorithm were utilized in this study to plan the motion path of the robot, and the two algorithms were compared with regard to convergence performance.

## Results

### The Algorithm Performance Analysis of the Improved Robot in Path Planning

**Figure 6:**
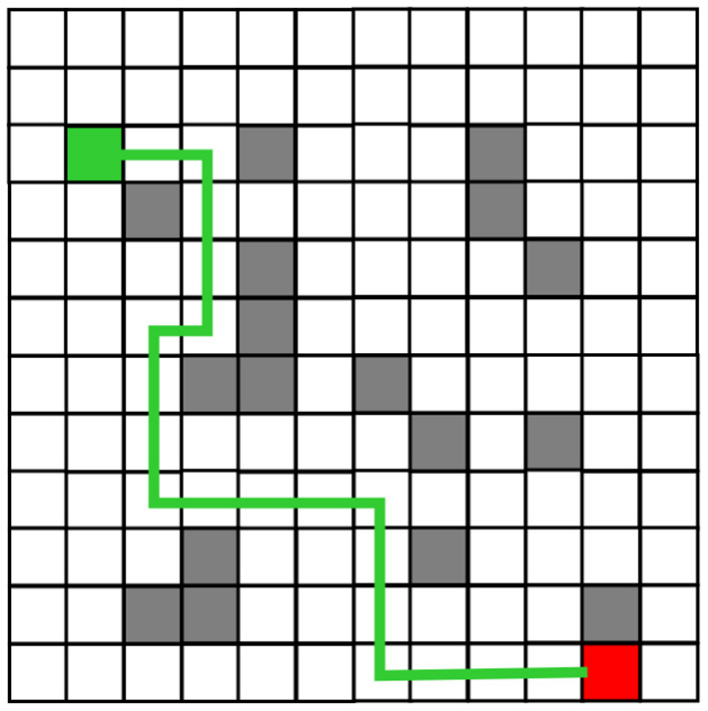
Obstacle Avoidance Path Planning of the Traditional Q-Learning Algorithm before Improvement.

**Figure 7:**
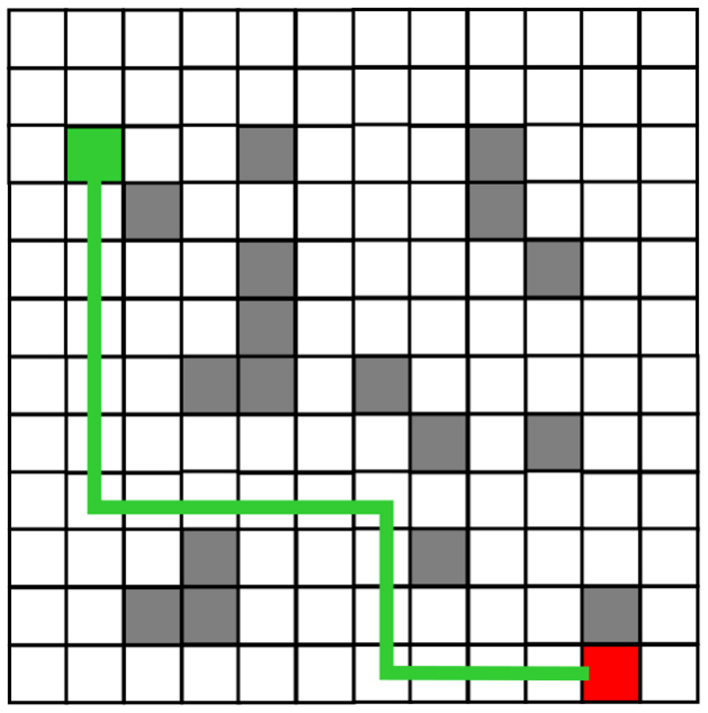
Obstacle Avoidance Path Planning of the Improved Q-Learning algorithm.

The robot planning path obtained from the simulation experiment of the traditional Q-Learning algorithm and the improved Q-Learning algorithm are shown in Figures 6, 7, respectively. Among these, black represents obstacles, and yellow grid represents the robot movement path. There are more obstacle avoidance path turning points obtained by the traditional Q-Learning algorithm. The obstacle avoidance path based on the improved algorithm is smoother, indicating that the improved Q-Learning algorithm is more suitable for robots to solve obstacle avoidance problems.

**Figure 8:**
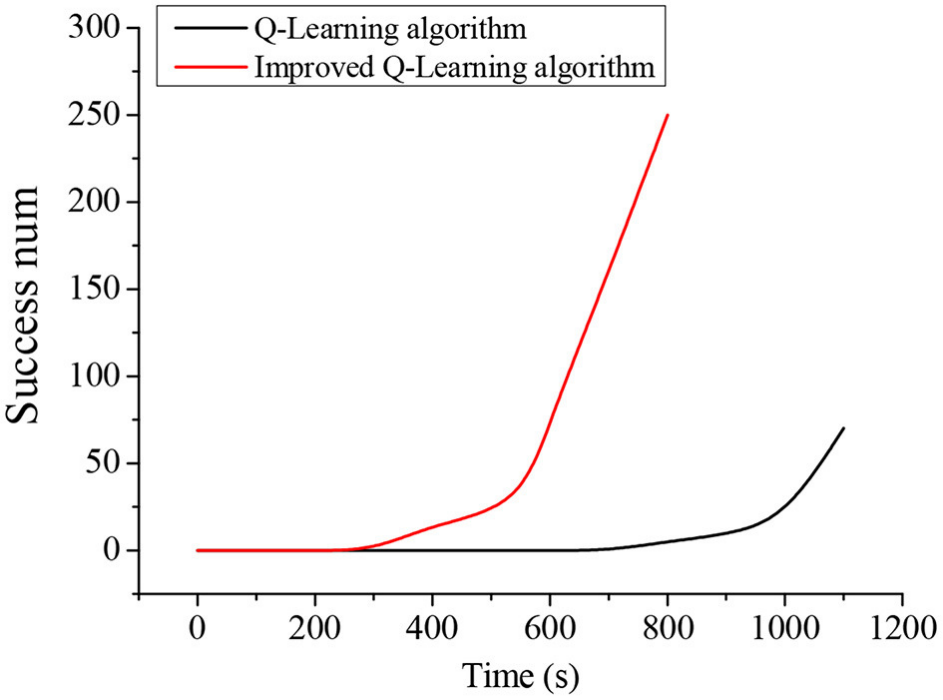
Comparison of the Convergence time of Q-Learning Algorithm before and after Improvement.

The time it takes for the traditional Q-Learning algorithm and the improved Q-Learning algorithm to converge in the training process is shown in Figure 8. It takes about 700 s for the robot to find the obstacle avoidance path to the target for the first time under the traditional Q-Learning algorithm, but the improved Q-Learning algorithm-based robot only needs about 250 s. Moreover, it is difficult for the traditional Q-Learning algorithm to find a path to the target in the initial training stage, while the improved Q-Learning algorithm can find a path to the target faster during the initial training.

### Obstacle Avoidance Function Test of the Robot

**Figure 9:**
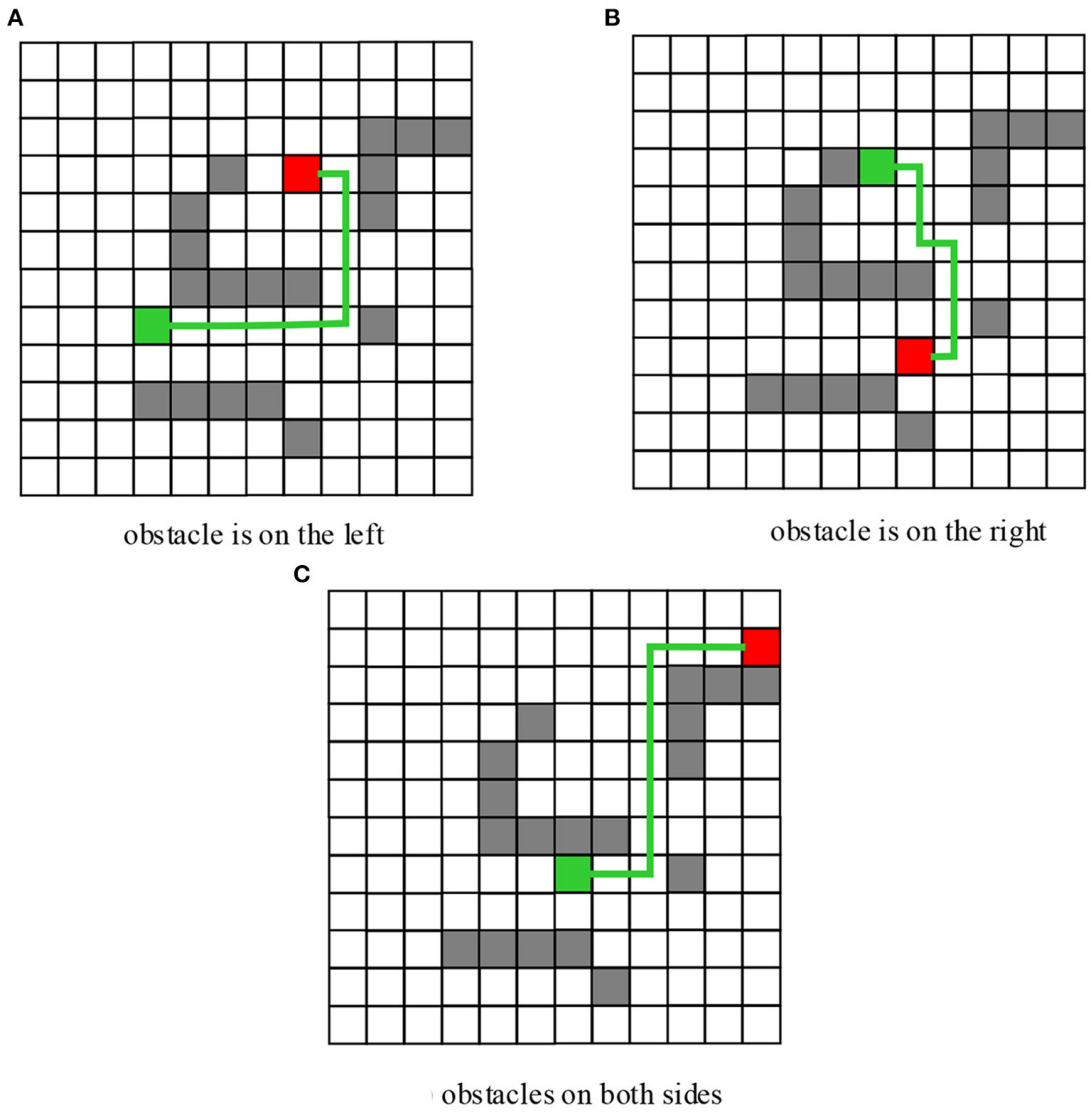
Simulation Results of Fuzzy Obstacle Avoidance of the Robot. **(A)** Obstacle is on the left. **(B)** Obstacle is on the right. **(C)** Obstacles on both sides.

Matlab programming was adopted to simulate obstacle avoidance behavior of the robot. The simulation results of the obstacle avoidance behavior when there are obstacles exist on the left, right, and left and right sides at the same time were shown in Figure 9. The starting heading angle was set to 60°, and the starting position and final target were randomly selected. According to the simulation experiment results, the basketball robot fuzzy controller established in this work can effectively avoid obstacles encountered during the robot movement. Moreover, since multiple fuzzy controllers were combined, the fuzzy rules were simplified, it was easier for the fuzzy control to implement, and the obtained motion trajectory curve was relatively smooth.

## Conclusion

In order to improve the practical adoption efficiency of basketball training strategies, and avoid chronic or acute injuries caused by blind training and collisions in basketball training, in this study, the scientific and effective training of athletes were improved based on the adoption of basketball intelligent robots. Firstly, according to the basic training actions in basketball training, the sport recognition in basketball training was analyzed, and in-depth analysis of the shooting action was implemented. In the training of the shooting action, a mathematical model was utilized to simulate the flying situation of the basketball without resistance, and the improved Q-Learning algorithm based on machine learning was proposed to plan the path of the sports robot. The improved algorithm supplemented a count threshold, and adopted the cumulative access times to the “state-action pair” <s, a> to determine the update of the Q value. In path planning, the fuzzy controller was applied to make the robot complete the approach to the target point and avoid obstacles at the same time. Sun et al. (2018) and Zheng and Liu (2020) showed that an optimized fuzzy control algorithm based on path planning could overcome the problem of excessively subjective fuzzy boundary selection and generate the optimal path, which was basically consistent with this research.

In the simulation experiment, the path planning of the robot through the improved machine learning algorithm shows that the improved Q-Learning algorithm can find a path to the target faster during initial training (Zheng and Ke, 2020). Moreover, the convergence time of the algorithm is considerably shorter than that of the traditional algorithm. In the subsequent obstacle avoidance performance test, the established basketball robot fuzzy controller can effectively avoid obstacles encountered during the robot movement. In addition, due to the combination of multiple fuzzy controllers, the fuzzy rules are simplified and the fuzzy control is easier to implement, making the obtained motion trajectory curve relatively smooth. In the research of the motion robot system, this work only discusses the motion planning of a single robot, but doesn't analyze the motion planning of the multi-robot system, which will be the focus of the next research.

## Data Availability Statement

The raw data supporting the conclusions of this article will be made available by the authors, without undue reservation.

## Author Contributions

All authors listed have made a substantial, direct and intellectual contribution to the work, and approved it for publication.

## Conflict of Interest

The authors declare that the research was conducted in the absence of any commercial or financial relationships that could be construed as a potential conflict of interest.
